# Viral Fragments in the Urine Proteome: New Clues to the Cause of Fever

**DOI:** 10.3390/biology14040318

**Published:** 2025-03-21

**Authors:** Minhui Yang, Yan Su, Chenyang Zhao, Youhe Gao

**Affiliations:** Gene Engineering Drug and Biotechnology Beijing Key Laboratory, College of Life Sciences, Beijing Normal University, Beijing 100875, China

**Keywords:** viral fragments in urine, proteomics, LC-MS/MS, fever of unknown origin

## Abstract

Background: To provide clues and a diagnostic basis for patients with fever of unknown origin through urinary proteomics analysis. Methods: For the first time, an attempt was made to conduct a full-library search for viruses in urine samples. Liquid chromatography–tandem mass spectrometry (LC-MS/MS) technology was employed to analyze the urinary proteomes of patients with fever of unknown origin, and to search for and identify viral protein fragments. In this study, there is no need to pre-determine the types of substances present in the samples. As long as the relevant sequences of viruses are available in the database, virus searches can be performed on the samples. Results: In the urine samples, multiple specific peptides from various viruses, such as the monkeypox virus, salivirus A, human herpesvirus 8 type P, Middle East respiratory syndrome-related coronavirus, rotavirus A, Orf virus (strain NZ2), human herpesvirus 2 (strain HG52), human adenovirus E serotype 4, influenza A virus, human coronavirus NL63, parainfluenza virus 5 (strain W3), Nipah virus, and hepatitis C virus genotype 2k (isolate VAT96), could be observed. It was found that the detection amounts of multiple viruses in febrile patients were much higher than those in the control group. Among them, the increase multiple of salivirus A was as high as more than 4200 times, and the increase multiples of multiple viral proteins were higher than 20 times. Conclusions: Viral fragments in urinary proteins can be reliably identified using mass spectrometry, which provides clues for the investigation of unexplained fever and may also be applied to the exploration of any unknown diseases.

## Simple Summary

Among the numerous causes of fever, accurately identifying the underlying etiology is essential for guiding clinical decisions. This study aimed to search for diagnostic clues for patients with fever of unknown origin by analyzing urinary proteins. The researchers collected urine samples from febrile patients and healthy individuals and detected viral fragments in them using liquid chromatography–tandem mass spectrometry technology. The results show that the detection levels of multiple viruses in the urine of febrile patients were much higher than those in healthy people. This indicates that viral fragments in urine can provide clues for investigating fever of unknown origin. In the future, it is expected to assist doctors in diagnosing fevers and other viral infectious diseases more quickly and accurately, thus safeguarding people’s health.

## 1. Introduction

Fever, a non-specific response to potential health threats, can be due to diverse causes, including bacterial, viral, fungal, and parasitic infections, inflammation, drug side effects, and tumors [1,2]. Given these varied causes, the accurate diagnosis by doctors during diagnosis and treatment is vital, as it is related to patient recovery and the effectiveness of the follow-up treatment.

According to epidemiological studies, fever of unknown origin accounts for approximately 1–3% of all outpatient and inpatient fevers [1]. The rapid identification of the exact cause of fever is not only helpful to ensure the accurate diagnosis of the disease, but also to start the treatment process in time, effectively curb the spread of the disease, improve treatment efficiency, and reduce medical costs. Through timely and accurate diagnosis, doctors can formulate targeted treatment plans to avoid unnecessary treatment and drug use, so as to protect patients’ health and improve the treatment effect [2]. However, currently, identifying the causes of fever of unknown origin remains a challenging problem. Despite the continuous advancements in medicine, accurately determining the root causes of fever in some febrile patients remains a formidable challenge [3]. Urine proteomics has shown great potential in the exploration and analysis of biomarkers [4]. Compared with other biological samples, urine has unique advantages: it is less strictly regulated by the physiological steady-state mechanism, so it can more sensitively capture the subtle biochemical fluctuations in the body [5]. In addition, urine can be obtained without professional collection means, and the collection process is not only non-invasive, but also convenient and fast, which makes it an ideal sample source for biomarker research [6,7]. Existing literature indicates that early related research can be traced back to the 1960s. In 1966, Ames and others examined the urine sediment of leukemia patients using an electron microscope, initiating the research on virus-like particles in urine [8]. In recent years, studies have proved that urine detection has potential in virus monitoring and diagnosis. For example, during the COVID-19 pandemic, scientists successfully detected the genetic material of neocoronavirus in the urine of patients, which provided the possibility of non-invasive and simple diagnostic methods. Similarly, the genetic material of the West Nile virus was also found in urine [9]. In addition, some scientists also detected SARS-CoV-2 nucleocapsid protein-derived peptide in urine samples [10]. These technologies have shown broad application prospects. Therefore, as a non-invasive and repeatable sampling method, urine detection is of great significance for the monitoring and early diagnosis of viral diseases, and is expected to play an increasingly important role in clinical practice [11,12].

In this study, we selected liquid chromatography–tandem mass spectrometry (LC-MS/MS) technology. It features high sensitivity and high precision, and can effectively detect both high-abundance and low-abundance proteins, thus presenting a more comprehensive view of the proteome [13]. We searched the full viral library of urine proteins of fever patients and normal people, and analyzed them in a fever group and a normal group. At the same time, because the causes of fever in each patient may be different, we also compared each patient with the normal group, in order to provide clues and a basis for the clinical diagnosis of fever through the differential proteins of viruses.

## 2. Materials and Methods

### 2.1. Collection of Patient Samples

The experimental materials were collected by Chenyang Zhao in the laboratory. The details are as follows: 11 urine samples from patients with fever at admission were collected from Beijing China–Japan Friendship Hospital, all of which were morning urine. The specific information is shown in Table 1. It should be noted that fever of unknown origin refers to a category of diseases in which patients have persistent fever for more than 3 weeks, with body temperatures exceeding 38.3 °C on multiple occasions. After at least one week of multiple examinations, the cause of the fever remains unclear. The body temperatures of such patients are not stable, and they are not always in a febrile state [14]. In this study, all patients were hospitalized due to fever of unknown origin. Although some patients were not in a febrile state at the time of sampling, they all met the basic characteristics of “fever of unknown origin” and had a fever (a body temperature ≥ 37.3 °C) upon admission to the hospital. It was just that at the specific time point of sampling, their body temperatures were within a relatively normal range. Their urine may have still retained biomarkers related to previous fevers, such as viral protein fragments. These samples were obtained in the laboratory department, without any patient identity information, and did not affect any treatment or recommend any clinical and auxiliary examinations. The purpose of the study is to obtain clues of fever, and the knowledge obtained is only used for research purposes. The patients’ temperature record is shown in Table 1. All participants signed an informed consent, and the study has been ethically approved by the China–Japan Friendship Hospital (No.: 2019-42-k30). A total of 8 cases were included in the healthy control group. Their body temperatures were all within the normal range, and none of them were patients with fever of unknown origin. They were medical staff recruited from Beijing You’an Hospital as reported in a previously published pre-print.

The raw data files of this experiment can be obtained under the project ID of the iProX dataset: IPX0002313003. URL: https://www.iprox.cn/page/PSV023.html (accessed on 3 January 2025).

### 2.2. Processing of Urine Samples

The collected urine samples were stored in a −80 °C refrigerator for future use. First, after defrosting the 4 mL urine sample, the cell debris was removed by centrifugal force at 12,000× *g* at 4 °C for 20 min. Then, the supernatant was mixed with 20 mM DTT and heated in a metal bath at 99.2 °C for 10 min. After cooling to room temperature, 50 mM IAA was added for light shielding reaction for 40 min. After the reaction, the sample was fully mixed with four times the volume of pre-cooled anhydrous ethanol and placed in a −20 °C refrigerator to precipitate the protein for 24 h. After precipitation, it was centrifuged at 4 °C, 10,000× *g* for 30 min, the supernatant was removed, the protein precipitation was blow dried, and appropriate lysate was added (8 mol/L urea, 2 mol/L thiourea, 50 mmol/L Tris, and 25 mmol/L DTT) to redissolve. The redissolved samples were centrifuged, the supernatant was retained, and the protein concentration was determined using the Bradford method.

Urinary protease cutting: the auxiliary enzyme cutting on the urinary protein membrane was performed using the filter-aided sample preparation (FASP) method. A total of 100 μg of urine protein was added to the filter membrane of a 10 kD ultrafiltration tube, and washed twice with an UA solution of 8 mol/L urea, 0.1 mol/L Tris-HCl (pH 8.5), and 25 mmol/L NH_4_HCO_3_ solution. Subsequently, trypsin was added at a ratio of 1:50 trypsin:protein and incubated at 37 °C overnight. After overnight incubation, the filtrate after enzymatic hydrolysis was collected by centrifugation, resulting in the polypeptide mixture. Finally, an Oasis HLB solid-phase extraction column was demineralized, vacuum dried, and stored at −80 °C.

### 2.3. LC-MS/MS Tandem Mass Spectrometry Analysis

The peptide was redissolved with 0.1% formic acid water, and the peptide concentration was diluted to 0.5 μg/μL. A total of 1 μg of the peptide sample was separated using a thermo Easy-nLC 1200 liquid phase system. The parameters were set as follows: elution time, 90 min; elution gradient phase A: 0.1% formic acid; phase B: 80% acetonitrile. The isolated peptides were detected by an Orbitrap fusion Lumos tribird mass spectrometer, and a data-independent acquisition mode was adopted.

During the analysis, 1 μg of peptides from each sample was loaded onto a C18 trap column and then separated using a reversed-phase analytical column at a flow rate of 1 μL/min with a 120 min gradient (buffer B: 2–6% for 1 min, 6–10% for 23 min, 10–20% for 67 min, 20–28% for 7 min, 28–95% for 20 min, 95–5% for 2 min). The LC settings were the same for both the DDA-MS and DIA-MS modes. In the DDA mode, ten fractions separated by a centrifugal column were analyzed using mass spectrometry to generate a spectral library. Full MS scans were acquired within the range of 350–1500 *m/z* with a resolution set at 120,000. MS/MS scans were acquired in the Orbitrap with a resolution of 30,000. Subsequently, individual urine samples were analyzed in DIA-MS mode. The variable isolation window of the DIA method consisted of 26 windows for DIA acquisition. The positive ion mode was set at 3000 V. The resolution of the full scan was set to 120,000 with an *m/z* range from 350 to 1200, and the resolution of the DIA scan was set to 30,000. To ensure data quality, pooled peptides from all samples were used to guarantee the stability of the instrument. A pooled DIA analysis was performed after every 7–9 samples as a technical replicate for quality control.

### 2.4. Data Analysis

Each polypeptide sample was taken for mass spectrometry in DIA mode, followed by data processing and analysis using Spectronaut X software (Biognosys, Biognosis, Switzerland) (Detailed information can be found in the Supplementary Materials). We downloaded the latest whole-virus protein data from UniProt to build the database, and then conducted a virus database search on the DIA raw data files of each sample. In this process, the parameters were set as follows: the FDR of the protein is 1%, and the FDR of the peptide is 1%.

## 3. Results

A total of 26 viral proteins were identified from these 19 urine protein samples after liquid chromatography–tandem mass spectrometry (LC-MS/MS) analysis, 13 of which had specific polypeptides. It should be noted that the retrieval error rate of the total protein is smaller than 1%, and the retrieval error rate of a single protein in each sample is smaller than 5%. Therefore, our protein retrieval results are very reliable, and the probability of all protein retrieval errors is only 1.2 × 10^−17^. Table 2 shows all the viral proteins with specific peptides detected.

Since the etiology of each febrile patient may be different, we adopted the one-to-many analysis method, that is, the comparative analysis of a patient and a group of healthy people’s samples to identify the differential proteins.

### 3.1. Analysis of Urinary Proteome in Single Patients

The urinary protein analysis of all patients with fever of unknown origin is shown in Table 3.

#### 3.1.1. Analysis of Urinary Proteome in Patient F1 

Compared with eight healthy samples, the samples of patient F1 showed that the detection amount of salivirus A was 4289 times higher than that of the control group, and the detection amount of rotavirus A was 16 times higher than that of the control group, indicating that the content of salivirus A and rotavirus A in the patient was higher than that of the normal people, which may be the cause of the fever.

#### 3.1.2. Analysis of Urinary Proteome in Patient F2 

Compared with eight healthy samples, the samples of patient F2 showed that the detection amount of salivirus A was 2962 times higher than that of the control group, and the detection amount of rotavirus A was 20 times higher than that of the control group. This indicates that the content of salivirus A and rotavirus A in the patient was higher than that of the normal people, which may be the cause of fever.

#### 3.1.3. Analysis of Urinary Proteome in Patient F3 

Compared with eight healthy samples, the samples of patient F3 showed that the detection amount of salivirus A was 18 times higher than that of the control group, and the detection amount of the monkeypox virus was 27 times higher than that of the control group. This indicates that the content of salivirus A and the monkeypox virus in the patient was higher than that of the normal people, which may be the cause of fever.

#### 3.1.4. Analysis of Urinary Proteome in Patient F4 

Compared with eight healthy samples, the samples of patient F4 showed that the detection amount of the monkeypox virus was seven times higher than that of the control group. This indicates that the content of the monkeypox virus in the patient was higher than that of the normal people, which may be the cause of fever.

#### 3.1.5. Analysis of Urinary Proteome in Patient F5 

Compared with eight healthy samples, the samples of patient F5 showed that the detection amount of the monkeypox virus was 6.9 times higher than that of the control group. This indicates that the content of the monkeypox virus in the patient was higher than that of the normal people, which may be the cause of fever.

#### 3.1.6. Analysis of Urinary Proteome in Patient F6 

Compared with eight healthy samples, the samples of patient F6 showed that the detection amount of the monkeypox virus was 21 times higher than that of the control group, the detection amount of salivirus A was 77 times higher than that of the control group, and the detection amount of the parainfluenza virus 5 was 16 times higher than that of the control group. This indicates that the content of these viruses in the patient was higher than that of the normal person, which may be the cause of fever.

#### 3.1.7. Analysis of Urinary Proteome in Patient F7 

Compared with eight healthy samples, patient F7’s samples showed no significant increase in viral proteins.

#### 3.1.8. Analysis of Urinary Proteome in Patient F8 

Compared with eight healthy samples, patient F8’s samples showed that the detection amount of Orf virus was 10 times higher than that of the control group. This indicates that the content of the Orf virus in the patient was higher than that of the normal people, which may be the cause of fever.

#### 3.1.9. Analysis of Urinary Proteome in Patient F9 

Compared with eight healthy samples, patient F9’s samples showed no significant increase in viral proteins.

**Figure 1:**
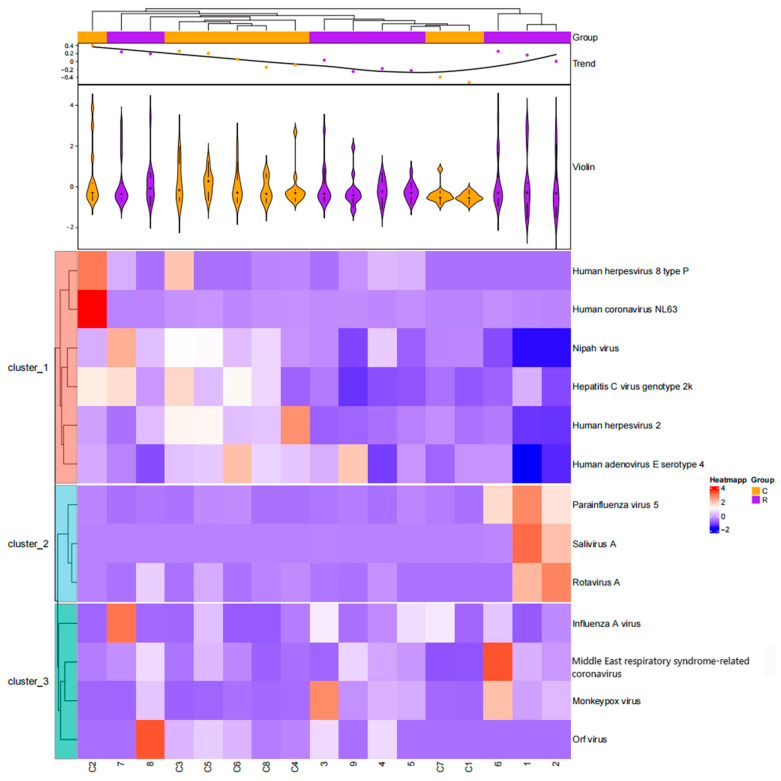
Heatmap analysis of viral proteins with specific peptides detected in all samples (C1–C8 represent healthy individuals, and 1–9 represent patients with fever of unknown origin).

The heatmap analysis of viral proteins with specific peptides detected in all samples is shown in Figure 1.

## 4. Discussion

By combining urine proteomics and one-to-many analysis, we found that there were obvious characteristics in the patients with fever examined: the amount of salivirus A and rotavirus A detected in the urine of patients F1 and F2 was notably higher than that in the control group. The amount of salivirus A and Monkeypox virus in the urine of patient F3 also increased markedly. Patients F4 and F5 showed a significant increase in the monkeypox virus. However, in the urine of patient F6, not only salivirus A and the monkeypox virus were detected, but also parainfluenza virus 5 was detected, and their detection levels were considerably higher than those in the control group. Finally, the Orf virus was detected in the urine of patient F8, and its order of magnitude was significantly higher than that in the control group.

The data in Table 3 of this study show that the urine of the healthy control group contained many peptides from viruses, and the levels of some viruses were higher than those in the urine of patients. However, existing studies have shown that healthy individuals may carry a variety of latent viruses (such as members of the herpesvirus family), or be exposed to non-pathogenic viruses in the environment (such as symbiotic viruses and animal-derived virus fragments) [15]. These viruses are in a silent state under the regulation of the host immune system and do not cause clinical symptoms, but they may still release trace amounts of viral protein fragments into bodily fluids through metabolic pathways [16,17]. In addition, the LC-MS/MS technology used in this study has extremely high sensitivity and can capture extremely low-abundance viral peptide fragments in healthy people, but these trace fragments may not have clinical pathogenic significance [18].

Although some viral peptide fragments were detected at low levels in the healthy group, the peptide abundance of specific viruses in the patient group shows a significant increase. For example, the abundance of salivirus A in patient F1 was 4289 times higher than that in the healthy group. This indicates that the abnormal proliferation of certain viruses in the patient group may be directly related to the cause of fever, while the low-level signal in the healthy group is more likely to reflect physiological or environmental exposure.

As a pioneering exploration, this study has certain limitations in terms of scale. It only included 11 febrile patients and 8 healthy controls. The small-scale cohort restricts the generalizability of the research findings, and it is necessary to conduct verification in larger-scale studies. In addition, the detection of viral fragments in this study relies entirely on LC-MS/MS technology and cannot be cross-validated with traditional virological methods such as PCR and serology.

Some viruses can release abundant protein peptides in urine. In order to effectively identify and track these emerging viruses, we can use de novo sequencing technology. This technology can accurately analyze the unique protein peptide sequence left by the virus in urine samples, so as to provide us with the molecular fingerprint of the new virus [19]. Based on these sequence data, we can build a database specifically for viruses in urine samples. This database will greatly facilitate researchers and clinicians, enabling them to quickly screen out potential virus information from urine samples and achieve rapid and accurate diagnosis [20]. In addition, the establishment of the database will also provide valuable resources for virological research and clinical treatment, and help us better understand the transmission mechanism, pathogenicity, and possible intervention strategies of the virus.

## 5. Conclusions

In this study, liquid chromatography–tandem mass spectrometry (LC-MS/MS) technology was used to analyze the urine protein of patients with fever, and a comprehensive virus library search was carried out. The results show that a variety of viral protein fragments were found in urine proteins, and the content of these protein fragments was significantly different from that of healthy people, such as salivirus A, the Orf virus, the monkeypox virus, parainfluenza virus 5, and other related proteins. This study confirmed the presence of viral proteins in the urine proteome. It provides new clues and ideas for the discovery of fever cases. It also provides new clues and ideas for the discovery of other viral infectious diseases.

## Figures and Tables

**Table 1:** Records of clinical characteristics of patients.

Patient Number	Sex	Age	Body Temperature/°C
F1	female	60	38.8
F2	female	81	38.4
F3	male	60	37.3
F4	male	73	37.8
F5	male	72	36.0
F6	male	64	36.6
F7	male	32	36.5
F8	male	71	36.8
F9	female	53	36.6
F10	male	88	36.8
F11	female	32	36.9

**Table 2:** All specific virus names and sequences.

Virus Name	Specific Peptide Sequence
Monkeypox virus	VGIAGLK
Salivirus A (isolate Human/Nigeria/NG-J1/2007)	ASVNSLLSGMVRTDVTR
Human herpesvirus 8 type P (isolate GK18)	PGVILLTK
Middle East respiratory syndrome-related coronavirus (isolate United Kingdom/H123990006/2012)	GTPVLQLK
Rotavirus A (isolate RVA/Human/Sweden/1076/1983/G2P2A [6])	SNISSISVWTDVSEQITGSSDSVRNISTQTSAISK
Orf virus (strain NZ2)	VVFTDLLIK
Human herpesvirus 2 (strain HG52)	PGAPAVPR
Human adenovirus E serotype 4	ALVLALR
Influenza A virus (strain A/Seal/Massachusetts/1/1980 H7N7)	NWTCTSITQNNTTLIENTYVNNTTVINK
Human coronavirus NL63	SGVIVFK
Parainfluenza virus 5 (strain W3)	LLLFCLR
Nipah virus	GALEIYK
Hepatitis C virus genotype 2k (isolate VAT96)	TIVAPDK

**Table 3:** Summary of urinary protein analyses of all patients.

Virus Name		Patient 1	Patient 2	Patient 3	Patient 4	Patient 5	Patient 6	Patient 7	Patient 8	Patient 9	Control
Monkeypox virus	MS quantification value	2,738,302.75	3,802,856.11	12,595,255.00	3,519,523.00	3,191,716.06	10,065,084.50	306,045.00	4,309,394.50	2,067,200.25	459,814.31 ± 171,566.22
	FC	5.96	8.27	27.39	7.65	6.94	21.89	0.67	9.37	4.50	
Salivirus A (isolate Human/Nigeria/NG-J1/2007)	MS quantification value	12,365,827.50	8,541,840.75	51,978.11	9382.64	0.00	223,416.01	304.56	0.00	0.00	2883.12 ± 3915.48
	FC	4289.04	2962.71	18.03	3.25	0.00	77.49	0.11	0.00	0.00	
Human herpesvirus 8 type P (isolate GK18)	MS quantification value	0.00	0.00	0.00	16,418.64	14,868.52	0.00	14,055.57	0.00	7850.39	16,092.09 ± 27,391.52
	FC	0.00	0.00	0.00	1.02	0.92	0.00	0.87	0.00	0.49	
Middle East respiratory syndrome-related coronavirus (isolate United Kingdom/H123990006/2012)	MS quantification value	3,382,280.50	2,808,975.00	1,555,474.63	3,117,784.50	2,753,179.38	10,178,591.50	2,562,041.25	4,424,595.25	4,309,881.75	1,935,426.25 ± 796,447.76
	FC	1.75	1.45	0.80	1.61	1.42	5.26	1.32	2.29	2.23	
Rotavirus A (isolate RVA/Human/Sweden/1076/1983/G2P2A [6])	MS quantification value	308,864.81	384,464.02	7309.42	41,916.95	0.00	0.00	0.00	123,758.55	0.00	19,126.31 ± 26,602.19
	FC	16.15	20.10	0.38	2.19	0.00	0.00	0.00	6.47	0.00	
Orf virus (strain NZ2)	MS quantification value	0.00	0.00	44,459.50	44,381.72	0.00	0.00	0.00	139,657.54	0.00	13,868.03 ± 15,622.77
	FC	0.00	0.00	3.21	3.20	0.00	0.00	0.00	10.07	0.00	
Human herpesvirus 2 (strain HG52)	MS quantification value	76,640.70	67,577.19	310,337.05	398,069.67	499,946.47	459,889.58	399,616.72	860,762.31	340,831.09	1,029,598.3 ± 538,036.5
	FC	0.07	0.07	0.30	0.39	0.49	0.45	0.39	0.84	0.33	
Human adenovirus E serotype 4	MS quantification value	85,753.20	123,977.40	344,412.55	157,878.00	303,869.02	299,514.16	270,137.13	178,808.48	603,185.19	379,161.97 ± 116,428.47
	FC	0.23	0.33	0.91	0.42	0.80	0.79	0.71	0.47	1.59	
Influenza A virus (strain A/Seal/Massachusetts/1/1980 H7N7)	MS quantification value	20,849.96	575,262.92	1,752,701.19	605,113.64	1,594,873.06	1,284,293.31	3,942,530.13	187,962.47	278,336.00	489,780.51 ± 630,111.83
	FC	0.04	1.17	3.58	1.24	3.26	2.62	8.05	0.38	0.57	
Human coronavirus NL63	MS quantification value	9238.28	6341.42	17,251.37	10,991.15	25,059.81	25,759.21	0.00	3385.14	19,464.56	99,478.54 ± 228,469.17
	FC	0.09	0.06	0.17	0.11	0.25	0.26	0.00	0.03	0.20	
Parainfluenza virus 5 (strain W3)	MS quantification value	156,499.11	98,796.96	3050.19	0.00	12,137.38	104,264.56	0.00	4075.66	6342.66	6302.82 ± 6816.75
	FC	24.83	15.68	0.48	0.00	1.93	16.54	0.00	0.65	1.01	
Nipah virus	MS quantification value	0.00	0.00	70,359.71	112,531.22	42,610.40	30,541.92	210,497.63	104,041.38	26,420.79	101,837.97 ± 31,989.54
	FC	0.00	0.00	0.69	1.11	0.42	0.30	2.07	1.02	0.26	
Hepatitis C virus genotype 2k (isolate VAT96)	MS quantification value	56,127.11	34,428.87	44,677.84	35,668.85	36,808.56	39,774.97	83,172.13	51,449.09	30,233.20	60,591.68 ± 18,991.17
	FC	0.93	0.57	0.74	0.59	0.61	0.66	1.37	0.85	0.50	

MS quantification value: the average of the MS quantification values from two MS injections for each patient. The control data are the average of the MS quantification values of eight healthy individuals ± standard deviation. FC (fold change): calculated as (patient mean intensity/control mean intensity).

## Data Availability

The mass spectrometry proteomics data of this experiment can be obtained under the project ID of the iProX dataset: IPX0002313003. URL: https://www.iprox.cn/page/PSV023.html (accessed on 3 January 2025).

## Supplementary Materials

The following supporting information can be downloaded at: https://www.mdpi.com/article/10.3390/biology14040318/s1: Table S1: The output of the Spectronaut analysis.
